# Pediatric Coccidioidomycosis Patients: Perceptions, Quality of Life and Psychosocial Factors

**DOI:** 10.3390/healthcare3030775

**Published:** 2015-08-28

**Authors:** Erin Mary Gaab, Fouzia Naeem

**Affiliations:** 1 Health Sciences Research Institute, University of California, Merced, CA 95343, USA; 2 Valley Children’s Hospital, 9300 Valley Children’s Pl, Madera, CA 93636, USA; E-Mail: fnaeem@VALLEYCHILDRENS.ORG

**Keywords:** pediatrics, coccidioidomycosis, valley fever, quality of life, children, families, coping, healthcare

## Abstract

Research investigating the effects of coccidioidomycosis (valley fever) on children and the psychosocial implications of this disease in general is lacking. This study reviews what is known about pediatric coccidioidomycosis patients. It documents the psychological functioning, quality of life, and illness perceptions of a sample of coccidioidomycosis patient families. Primary caregivers of pediatric patients and patients from a major hospital in the San Joaquin Valley of California were interviewed regarding their perceptions of disease detection, access to care and the patient/family experience.

## 1. Introduction

### 1.1. Coccidioidomycosis

Coccidioidomycosis (valley fever) is an infection caused by the fungus Coccidioides immitis/posadasii, which is endemic throughout the southwestern United States [1,2], especially in the southern San Joaquin Valley of Central California [3]. The illness is contracted by the inhalation of arthroconidia (fungal spores). Coccidioidomycosis affects people across the lifespan; the youngest early case reported in an early study was 15 months old and the eldest was 69 years [4]. About one quarter of the initial cases studied fell into the 10–19-year-old age group. However, this data may be skewed by a change in the method of detecting coccidioidomycosis. Our understanding of coccidioidomycosis diagnosis and treatment is still developing. In California’s four highest-incidence counties, 3.9 children under the age of 14 were hospitalized, compared to 57.2 of those between the ages of 50 and 69 years old (per 100,000 residents) between 1997 and 2002 [5]. In more recent years, the highest average annual incidence rate has been reported among 45–54-year-olds (14.8 per 100,000) [6], with the lowest incidences in children and those over 85. In addition to people over 60 years old, people of Hispanic, African-American, and Filipino origin are at increased risk for developing severe disease. However, recent reports of demographic data are incomplete. Due to a significant amount of missing California data (44.4% of incidence rates by race/ethnicity) in a report by the Center for Infectious Diseases, incidence rates by ethnicity were not reported. The lack of complete data and low rates of reported infection in children may be expected to translate into a gap in terms of the provision of comprehensive care to this vulnerable group.

### 1.2. Diagnosing Coccidioidomycosis

The first major publication about coccidioidomycosis in the USA was printed in 1940 [4], though the illness was discovered prior to that date. Up to 60% of people infected with coccidioidomycosis do not express any symptoms [7], so diagnosing and treating valley fever patients may be difficult for healthcare providers. However, some of those diagnosed develop a very serious, prolonged form of the disease when the fungus spreads to their lymph nodes, bones, joints, brain and other organs [7]. Symptomatic infection presents in a variety of ways. Most commonly, coccidioidomycosis symptoms are flu-like (with or without chest film) and may include a fever, headache, cough, or myalgia. The flu-like symptoms generally develop after a nine to 13 day incubation period. Patients with coccidioidomycosis may also develop pneumonia or meningitis or a more serious disease. If caught during an earlier stage, coccidioidomycosis spending can be curtailed by decreasing patient anxiety and potentially preventing the spread of the infection. Unfortunately, coccidioidomycosis is not always diagnosed. When doctors began recognizing the coccidioidomycosis, it was reportedly often misdiagnosed as a communicable disease such as influenza, pneumonia, measles, tuberculosis, poliomyelitis, typhoid fever or syphilis [4]. Over 100,000 annual cases of coccidioidomycosis were reported in recent years, making it a nationally reportable disease in 1995 [3]. However, the disease is still said to be underreported [8]. This may be due to infected individuals who are asymptomatic or exhibit flu-like symptoms not seeking medical evaluation [1]. Under-evaluation may also contribute to an underestimate of annually reported coccidioidomycosis cases.

As with all public health issues, addressing issues in coccidioidomycosis has financial implications. Diagnosing and treating coccidioidomycosis requires community resources. A dramatic increase of coccidioidomycosis in Kern County, California the early 1990s [9] was estimated to cost over $ 66 million in direct medical expenses and time lost from work in Kern County alone. Although children are not working, time spent away from their classrooms and decreased productivity at school due to fatigue and other symptoms may yield long-term implications for the economy and society at large.

### 1.3. Children with Coccidioidomycosis

Little has been written about children with coccidioidomycosis [10,11,12,13]. Though early research demonstrates a higher rate of testing positive for coccidioidomycosis in children [4], perhaps due to more population-based testing in children, it is believed that there may be a lower rate of disseminated coccidioidomycosis in children than adults [7].

Like adults, most children who contract coccidioidomycosis are able to resolve the infection without treatment [14]. However, when a child’s immune system is unable to control the infection, it can spread locally or disseminate, most often to the skin, lymph nodes, meninges and/or skeletal systems. Disseminated infections also appear to be more frequent in adults than in children [15]. Most hospitalized pediatric patients present with fever, fatigue, cough and localized pain, usually in the chest or back [13].

Newborns are also at risk of developing coccidioidomycosis. Current evidence indicates that neonatal infection is uncommon and is most likely acquired during delivery [16,17]. Between 1950 and 1967, there were 26 cases of coccidioidomycosis among the 33,736 live births at Kern County General Hospital, yielding an incidence rate of 7.7 per 10,000 pregnancies [18]. Although pregnancy is a risk factor for the development of severe and disseminated coccidioidomycosis in the mother, especially when acquired during late pregnancy, there is no recent evidence of fetal wastage or prematurity in patients’ newborns [19].

A substantial proportion of Infectious Diseases practice in the largest pediatric hospital in Central California is comprised of coccidioidomycosis patients [13]. Anecdotally, doctors report that, in many cases, disseminated pediatric coccidioidomycosis patients experience fatigue and other symptoms that limit them from some activities [20]. Research is needed to characterize the profile, effects and implications of coccidioidomycosis in children. 

### 1.4. Psychosocial Aspects of Coccidioidomycosis

Little has been written about the psychosocial implications of coccidioidomycosis. To our knowledge, only one published study looks beyond strictly physical symptoms, disease incidence and biological underpinnings of the cocci fungus. Hoffman Snyder [21] examined cognitive dysfunction as a less frequent first symptom of coccidioidomycosis (in adults) that may be diagnosed as dementia. The author concluded that if undiagnosed or misdiagnosed, coccidioidomycosis may result in death.

To our knowledge, there are no studies of the quality of life or well-being of coccidioidomycosis patients in adults or children [22]. Disease-related stress and long term psychological effects remain unknown.

### 1.5. Specific Aims

This research is intended to increase knowledge and understanding of psychosocial issues faced by a group of families with children with symptomatic coccidioidomycosis. Specifically, we aim to document psychological functioning, quality of life, and illness perceptions of coccidioidomycosis patients and their families, emphasizing families’ questions around detection, access to care and outcomes. This research aims to explore how families in California’s Central Valley may cope with San Joaquin Valley fever in order to set priorities for better serving them.

## 2. Research Design

### 2.1. Methods

The study sample consisted of pediatric patients receiving care for coccidioidomycosis at a pediatric hospital in the San Joaquin Valley. Potential participants were identified by staff at the pediatric hospital in the San Joaquin Valley as new or existing patients and were referred to the hospital. The referred patients (outpatients and inpatients) were invited to participate by their primary care physicians. Children over six years of age diagnosed with coccidioidomycosis (or primary caregiver only of children with coccidioidomycosis of age three or older) were included. All participants were functioning at a developmental level sufficient for completion of interviews (for primary caregivers), KIDSCREEN-27, Child Illness Perceptions Questionnaire (CIPQ), KidsCope and drawing tasks.

Participants were given information about the study by their primary care physician at a pediatric hospital in the San Joaquin Valley. They were then invited to take part in the research from a researcher at the University of California, Merced (UCM). Participants were required to speak English, Spanish, Mandarin or Hmong.

### 2.2. Procedures

At study enrollment, after going over information about the study and having the opportunity to ask questions, participants were asked to sign consent and assent forms. The Principal Investigator (PI), accompanied by one or two Spanish-speaking research assistants, sought written informed consent using standard IRB-approved forms (HSC-1093), documenting the purpose, risks, benefits, and alternatives to participation in the study. Written informed assent was also sought from minors using additional approved forms. The interviews were then administered to participating primary caregivers, who consented to an audio recording of their interview. After the primary caregiver completed the interview (in English or Spanish), they were asked to fill out a questionnaire as their child was assisted by the researcher in responding to structured questionnaires. Translation services were provided by trained UCM students for the interview and survey questions, who observed and assisted with the analysis of all interviews to ensure consistency and participant safety. Raw data was stored in a locked file cabinet at the principal investigator’s office at UCM.

Consenting primary caregivers filled out questionnaires asking about basic demographic and illness data; their experiences with diagnosis, treatment, and healthcare; and their child’s comorbidities and overall health. An investigator (the PI or a research assistant trained and supervised by the PI) assisted participating primary caregivers as needed in understanding the questionnaires and completing the measures. If parents consented to having their children with coccidioidomycosis (age 6 and up) participate and children gave their assent, children were asked to rate their overall health and give measures of quality of life (KIDSCREEN-27), illness perceptions (a drawing task and CIPQ), and coping skills (Kidcope). While each primary caregiver completed the questionnaire with the aid of a research assistant, data was collected from the child by the PI.

Before enrollment, the measures were piloted on one English family and one Spanish family with children being treated for coccidioidomycosis before initiating the full project to prospective patients. Each assessment was available in Spanish and English. Mandarin and Hmong translators were available but were not requested by any participants.

### 2.3. Data Collection from Primary Caregivers

In order to document some of the perceptions of impaired functioning, fatigue and other symptoms and to understand general psychosocial issues faced by families of a child receiving treatment for coccidioidomycosis, consenting primary caregivers reported on their experiences orally. They answered semi-structured interview questions followed by 17-page questionnaires. These tools allowed primary caregivers to document their current family demographics; their experiences with diagnosis, treatment, and healthcare; and their children’s comorbidities and overall health. These constructs were measured using adapted versions of survey questions used to assess patients with chronic diseases and modified by a team of public health researchers at UCM and Pediatric Infectious Disease physicians to minimize bias and ensure that results might be used proactively. The following questions were not asked directly of participants, but outline the basic process of inquiry:

- ***Detection***
  (1) How (and where/by whom) is coccidioidomycosis detected by child patients, their families and their healthcare systems?
  (2) What causes delays in diagnosis?
- ***Access to Care***
  (3) What barriers impede patients and families’ access to care/treatment?
  (4) What (information, support, etc.) do patients and families feel that they need?
- ***Experience***
  (5) What difficulties (physical, psychosocial, etc.) do patients and families face as a result of having pediatric coccidioidomycosis?
  (6) What myths about coccidioidomycosis do patients and families currently hold?

After each interview concluded, it was transcribed by a research assistant who was present at the interview within one week. Their initial impressions, ideas and biases were recorded in memos written during and after the transcription process. All interviews were initially transcribed orthographically. Once complete, the transcription was checked by the research assistant for accuracy and notation symbols were added, such as ((laughs)) for laughter, (.) for each second-long pause between speech segments, (..) for each two-second pause, (..) for each three-second pause, *etc*. Analysis began once all interviews were recorded transcribed and (if necessary) translated. English to Spanish back translation methods were used in the design, data collection and data analysis phases of the research. The interview analysis was conducted over a six-month period and led by the PI. Inductive thematic analysis was employed, whereby line-by-line descriptive codes were used to label the data before themes were drawn from the data. The physician was informed of the results, but not involved in the data analysis, in order to minimize bias.

### 2.4. Quality of Life Scales with Pediatric Patients

It is fairly well known that parent-proxy reports of quality of life differ depending on who assesses the quality of life [22] with child reports being considered more accurate. Children were given several opportunities to report their perceived quality of life. They were first given a researcher-created scale typically used with adult populations. As a warm-up activity, the researchers asked children to point to their overall quality of life on a thermometer-like scale. The KIDSCREEN-27 [23], a Health-Related Quality of Life instrument, was developed for use in international pediatric populations. The KIDSCREEN-27 was administered to patients receiving treatment for coccidioidomycosis.

Research assistants supervised by the PI used Microsoft Excel (Microsoft, Redmond, WA, USA, 2010) to enter the raw data. Both Excel and SPSS (IBM Corp., Armonk, NY, USA, 2013) were used to note frequencies and generate graphs from the data. Due to the small number of participants, only frequencies were examined and salient results were reported.

### 2.5. Drawing Task with Pediatric Patients

A drawing task served as a less-structured platform for the exploration of themes generated from their own and others’ qualitative and quantitative data. Patients were given the following instructions: “Please draw a picture of what you think your body looked like before you were diagnosed with valley fever (drawing 1) and another picture of what you think your body looks like during treatment for valley fever (drawing 2). We are interested in what you think has happened to your body.” When finished, children were asked to describe what they drew. The interviewer recorded children’s comments about their drawings below each sketch. Drawings were used as both a data generation tool and as data themselves. They were used primarily to check the accuracy of themes generated from the other qualitative and quantitative data and served as another set of results for data triangulation.

### 2.6. Child Illness Perception Questionnaire with Pediatric Patients

Illness perceptions are cognitive representations of illness held by patients that influence patients’ health behaviors [24]. The Illness Perception Questionnaire (IPQ), created by Weinman *et al.* [24], is one of the most common tools used to measure cognitive and emotional perceptions of illnesses. This has been used in adult populations and was recently adapted for use with the child population [25]. The scale measures patients’ attributions of the causes of their illness to factors such as family history, diet, bad medical care, stress, their own behavior, chance and germs. By changing response scales from the likert-style “strongly agree” to “strongly disagree” to a dichotomous true/false response, prior studies [25] have confirmed the use of a dichotomous IPQ scale to elicit responses as the most appropriate, user-friendly method for accessing answers from patients and physicians.

The measurement scale of the Child Illness Perceptions Questionnaire (CIPQ) [25] has been used to assess several illness groups including children with asthma, cancer, eczema, and cystic fibrosis. Since the CIPQ we used has been adapted for use with child population with eczema/asthma (which are known to be stress-related), it has limited applicability within our sample of pediatric cocci populations. The CIPQ was used to assess children’s illness beliefs concerning the timeline, control/cure, consequences, and causes of their illness using true/false responses to questions.

Microsoft Excel was used to organize the raw data before SPSS was used to determine frequencies and create graphs for both the CIPQ and coping scale results.

### 2.7. Child Coping Scale with Pediatric Patients

Several measures exist to evaluate how children cope with illness and other stressors. It is assumed that coccidioidomycosis is a stressor, considering its sometimes chronic nature, known symptoms, and prolonged treatment schedule. The Kidcope is for children between seven and 18-years-old [26,27]. Other slightly less inclusive scales include the Responses to Stress Questionnaire (RSQ) [28] and the Coping Strategies Questionnaire [29]. The more inclusive measure in terms of age and disease stressor, the Kidcope, was selected to measure children’s coping with coccidioidomycosis in this research. The Kidcope is a 15-item brief coping checklist [27]. It includes the coping strategies of blaming others, cognitive restructuring, distraction, emotional expression, problem-solving, resignation, self-criticism, social support, social withdrawal, and wishful thinking.

### 2.8. Triangulation between Data from Primary Caregivers and Pediatric Patients

Themes from the caregiver interviews and surveys were cross-examined against the drawings and KIDSCREEN, CIPQ, and KidsCope results to form a broad picture of the experiences of families with pediatric coccidioidomycosis patients. This was conducted through simultaneous analyses of the visual and qualitative data and consistent communication between each analyst (trained in qualitative methods) about potential themes. As mentioned, qualitative data was transcribed, memos were written and themes were generated and refined by 15 research assistants and analyzed by the PI and colleagues at the university, decreasing bias from involved healthcare providers. A variety of qualitative and graphed quantitative analyses were performed using NVivo qualitative data analysis software, Version 10 (QSR International Pty Ltd., London, UK, 2012), Microsoft Excel, and SPSS software for data organization and analysis. The variety of tools used to collect and analyze this project maximized the chance of generating a range of responses from our data which were then synthesized through triangulation. Braun and Clarke’s method of inductive thematic analysis was used to analyze the qualitative data [30]. However, since the sample recruited is small, only salient qualitative results and visual analyses of quantitative data are reported.

## 3. Results

### 3.1. Participants

Twenty-two primary caregivers and 15 children with coccidioidomycosis participated in the research. This purposive sample represents more than half of the 42 potential participants contacted by the research team (since a portion of the children were too young to participate). The strong rate of participants consenting may reflect the researchers’ willingness to meet participants at the location of their choice: the hospital, home or places of work. Those who refused may have done so because of the substantial time commitment. One primary caregiver and one child from each of the 22 families was allowed to participate. The families included children who ranged in age from three to 18, though the child participants were six and older. The study sample reflected the approximate makeup of the coccidioidomycosis patient population served by a pediatric hospital in the San Joaquin Valley with respect to age, sex and ethnicity. Most of the primary caregivers who participated in the interviews were female. Among children in this county, approximately 59% are Latino, 21% are white, 10% are Asian/Pacific Islander, and 5% are African American/Black [31]. Figures in the surrounding counties of the San Joaquin Valley are similar. Comorbidities were similar to those of the target population, with several patients who also reported health concerns related to asthma and obesity. This small dataset (composed of about 10% of the Infection Disease patient population at a children’s hospital in the San Joaquin Valley) provides a general picture of how living with coccidioidomycosis is perceived by some children and families.

### 3.2. Exploratory Interviews

**Figure 1:**
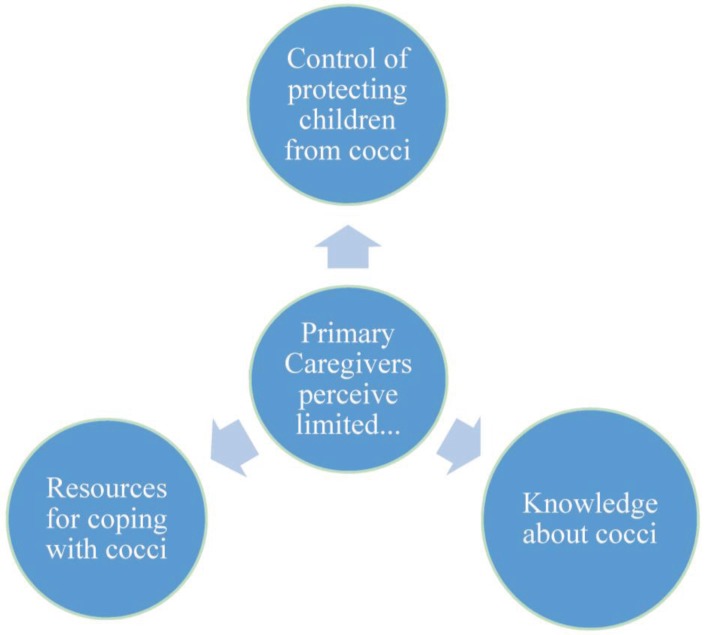
Perceptions of primary caregivers of children with coccidioidomycosis.

The following themes emerged from the qualitative interviews with patients’ primary caregivers: deficient knowledge about coccidioidomycosis, a lack of control over protecting children from valley fever, and limited resources for coping with the illness. These themes are illustrated in Figure 1.

#### 3.2.1. Lack of Control

A lack of control over preventing coccidioidomycosis and living with it was expressed by many primary caregivers, often resulting in emotional strain. There was little that most caregivers noted that they could do to prevent valley fever. Despite this, feelings of guilt were expressed by most of them from not being able to prevent or relieve children’s symptoms. It is clear that having a child with coccidioidomycosis can cause major strain on families, including less time with siblings as well as expressed out-of-control emotions, such as guilt and fear.

Helplessness over controlling the illness was expressed. The treatments sometimes were described as being difficult for children to tolerate (and parents to witness): “They went over everything to a T with me on what was going to happen with that medication I knew exactly what that was gonna do to my kid (…) but I also knew we didn’t have a choice (…) and I knew that we were pouring straight toxin into my kid.”—Parent A.

The complex needs of these children often took time from time that parents might otherwise be spending with other siblings: “I struggled (…) to find a place where I could leave [the patients’ siblings] during the time they were going to school.”

Another primary caregiver expressed her difficulty managing her own emotions while coping with her child’s orphan disease: “You know the amount of stress and loneliness and fear and (….) is astronomical it really is to sit in that room and go through what I went through watch a child going through all this and being stuck over again and (…) and having no one really there he couldn’t deal with it. His stress level had to send him somewhere else and I was very angry at that point because he wasn’t there.”—Parent B.

Another mother spoke about her guilt: “I (…) cry when she come to the house …Was it my fault? …What happened to her? Why? Why? Why? (…) I was like calm down crying and tears like, ‘Why? Why? What’s wrong? Was I a bad mom?’”—Parent C.

Managing the treatments themselves was also difficult for many: “the right...dosage and this one here, and this one there, I mean it was (…) I had ten different pill bottles going, okay get this one now, three of these tomorrow (…) It was insane.”—Parent D.

Another described her own suffering on watching her child undergo treatment: “....it seems like the whole world was falling like into like say like oh no I ugh I wanna pull my hairs out cause I don’t know what to do like I wanted to like take his pain away like for me to have it.”—Parent E.

#### 3.2.2. Lack of Knowledge

Most primary caregivers expressed a lack of knowledge about valley fever. They commented on the need for patients, healthcare providers (outside the infectious disease clinic), and the public to learn more about coccidioidomycosis. Several primary caregivers described inaccurate beliefs about valley fever developing from exposure to fertilizers, pesticides and other chemicals used for farming and/or specific waste sites. They expressed concern over this lack of knowledge resulting in misdiagnoses, increased use of the healthcare system (multiple visits at multiple sites) and decreased trust in healthcare providers. Some parents felt misinformed and expressed a need for more information about the cause of valley fever. A few wanted an explanation of why the symptoms vary between individuals with coccidioidomycosis (the latter of which is still unknown in the literature).

One primary caregiver expressed having never heard of valley fever until recently: “...I hadn’t (.) hadn’t heard that of valley fever (.) until we came here...”—Parent F.

Many participants expressed medically inaccurate beliefs regarding the causes of valley fever. As stated, several mentioned the cause of valley fever as chemical fertilizers (a misconception prevalent in our sample): “is in our ground (.) and I don’t think that it’s naturally in there I think that it’s manmade in there with all the crap that we spray (.) and all the chemicals that go in the ground and all the I mean this valley is a heavy farming community and we spray a lot crap out here.”—Parent F.

Another still had no idea how her son had developed the illness: “…No, I honestly don’t know why he got it, the illness. And no one ever told me why he got it. Just from talking with neighbors they would say it was because his defenses were very low.”—Parent E.

Some of the participants asked more questions of the researchers than the researchers asked of them, expressing confusion over multiple aspects of the illness: “Still to this day [I] don’t really understand a damn thing about Valley Fever … Half the time … you’re dealing with doctors that don’t know the area (.) don’t understand the area.”—Parent G.

Coccidioidomycosis was described as a mythical illness by one participant:

I mean I heard about it [Valley Fever] but it’s like this myth through this valley one person will says this little thing about it or somebody nobody really, really discussed (.) you know and especially in the hospital [beyond the specialist clinic]; you ask them they have no clue what valley fever really even is...you hear like I said it’s a myth you’d hear someone say ‘oh my great uncle 20 years ago got it, or this that and the other. Never really anything significant.

—Parent H.

#### 3.2.3. Limited Resources

Most primary caregivers spoke about a lack of informational, emotional and practical support for families dealing with pediatric valley fever. Communicating information about valley fever from healthcare providers to patients’ families and from families to others was presented by several families as a challenge. Language barriers (most commonly Spanish to English or vice versa) exist. Several primary caregivers mentioned the need for a support group to assist families in addressing emotional and information challenges, some of which are supported by quotes from the previous themes: “The doctors were great, the hospital was great you know all that stuff was wonderful but not having that support, that emotional support, from parent to parent going through this somebody else truly understands where I’m at.”—Parent B.

Transportation to healthcare facilities is a financial and time barrier to care for several families as well. Financial strain was described by several parents: “They like cost I believe like (.) the two it was sixty pills for almost like a thousand dollars? (.) and Medi-Cal wouldn’t cover it.”—Parent I.

Another parent described the financial strain as challenging:

“I was you know disability family disability doesn’t pay (laughs) so the amount of what I actually got on my paycheck was it a financial strain? Oh hell yeah in between driving um (.) Dad coming back and forth the whole time I was up there me not having my wages while I was up there um food because food at children’s is expensive you know hers is taken care of mines not um so I mean that’s so I was lucky they’re were able to do the ’fridgerator for me and there and stuff so it did help I’d go to the grocery store and pick up a few things here and there but um man the cost was bad (.) it was hard.”

—Parent C.

A need for more informational, emotional, and practical support was expressed by most parents of pediatric coccidioidomycosis patients who participated in the interviews.

Overall, the experiences of children and families dealing with coccidioidomycosis were strained by a lack of knowledge about coccidioidomycosis, a lack of control over protecting children from the illness, and a lack of resources for families coping with it.

### 3.3. Primary Caregiver Survey Results

Primary caregivers reported their experiences with diagnosis, treatment, and healthcare and their children’s overall health on surveys after they were interviewed. Discussing each question answered by primary caregivers goes beyond the scope of this article. However, a few salient results were generated. A variety of symptoms and levels of impact on children’s lives were reported.

Primary caregivers first noticed the wide range of main symptoms (from severe shortness of breath to mild rashes to an absence of symptoms) and their children’s overall health status at that time also ranged widely (from poor to good at the time the first symptom was noticed). Less than one quarter of the families interviewed reported that coccidioidomycosis was suggested as a potential cause of their child’s first symptom(s) on their first visit to a healthcare provider. Most parents stated that the child’s fever was the worst symptom of coccidioidomycosis at its worst point in the child’s illness trajectory, although fatigue, chest pain, pulmonary symptoms, weight loss, vomiting, pain, and headache were also mentioned. About one quarter of parents stated that their child’s health was “good” at the worse time during their child’s treatment. Most primary caregivers believed that the illness had a minor or moderate impact on their child’s schooling, but responses ranged from “no impact” to “severe impact”. Most noted at least a mild impact on their child’s social development/interactions with friends. Several categorized the impact on their family (including needing to take time off work) as “severe”.

With regard to getting information about the illness, most primary caregivers initially indicated that they had received most of the general information they needed, but still had some unanswered questions. Almost half of the primary caregivers, however, felt that they needed information about the treatment of coccidioidomycosis and did not receive the information they needed. A notable portion of primary caregivers also felt that they needed information about the emotional and social effects of coccidioidomycosis on their child but did not receive that information. A few also noted that they required practical support: extra help with paying medical bills, help at home, help with childcare, and help understanding services that might be available to them.

Most of the primary caregivers (15/22) indicated that they knew where their child was exposed to coccidioidomycosis. Many of the areas named were in agricultural settings where children would generally inhabit (at home, at school, on a ranch, on a construction site, at a park, *etc.*). However, a large number (7/22) of others did not know where the child was exposed. When asked how likely it was that their child was exposed to the illness from a construction site, several parents stated “not at all likely”, despite the informed suspicion of several others living in the same area alleged to be home to the fungi. One participant expressed a belief that coccidioidomycosis came from pollen and plant matter. Partly because of the mystery of how the illness is contracted, every primary caregiver expressed that asking a doctor to test for coccidioidomycosis is important (most indicating that it is “very important”). Comments about a range of symptoms, resulting impacts, and levels of knowledge about coccidioidomycosis were generated from the primary caregivers.

### 3.4. Children’s Quality Life Scale Results

In terms of physical activities and health, every child interviewed using the KIDSCREEN-27 [22] stated that (in general) their health was good, very good or excellent. Almost half of them (seven of the 15 child participants) reported that over the last week, they “always” felt “full of energy” and they all reported feeling moderately, very or extremely “fit and well”, contrary to parent-reported fatigue. When addressing questions of their general mood and feelings, a substantial portion (6) of the children reported “feeling so bad, [they] didn’t want to do anything”, which may be a more typical sign of fatigue in pediatric populations previously mentioned by medical doctors.

In terms of family and free time, several patients (4) with coccidioidomycosis stated that they “never” or “almost never” “had enough money to do the same things as [their] friends”, though almost half (7) reported that they “had enough money for expenses”. Since nearly half (6) reported not always having enough money for expenses, a feeling of financial strain may be affecting pediatric coccidioidomycosis patients as well. Most (13) also reported having “fun with [their] friends” most or all of the time. Most children also reported feeling supported by peers and stated their comfort in the academic setting at levels typical of developing children.

**Figure 2:**
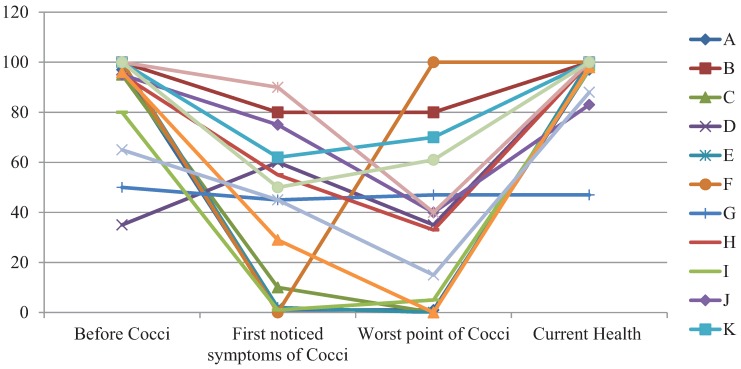
Self-reported Quality of Life of children with coccidioidomycosis.

On a Quality of Life Scale, the majority of pediatric patients tended to show more positive health now than before or during their valley fever diagnosis. Although this activity was intended as a warmup, it reflected the positive attitudes of the children with coccidioidomycosis sampled. The children reported a variety of perceptions of their quality of life during different times over their illness trajectory (Figure 2).

### 3.5. Children’s Drawing Task Results

**Figure 3:**
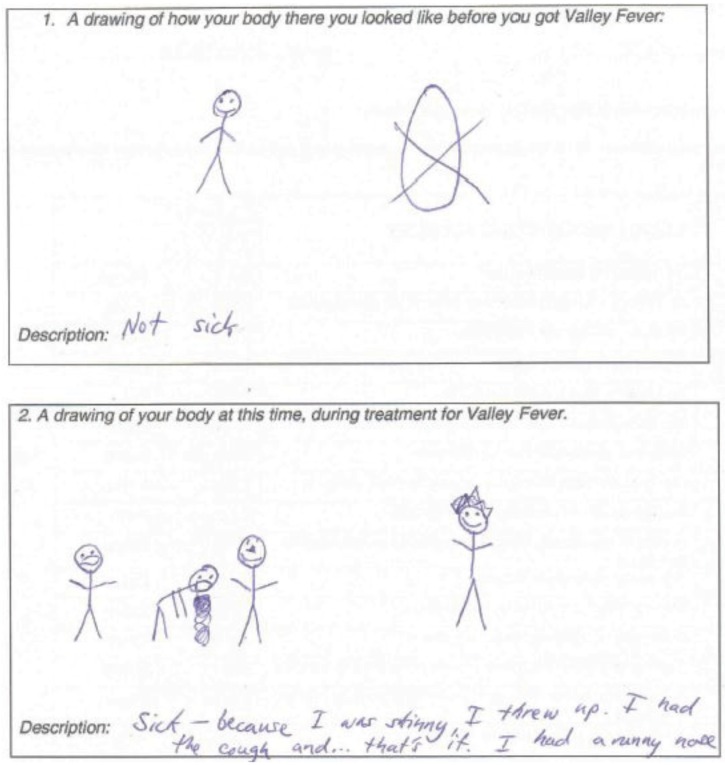
Drawing of a child with coccidioidomycosis with a focus on physical symptoms.

**Figure 4:**
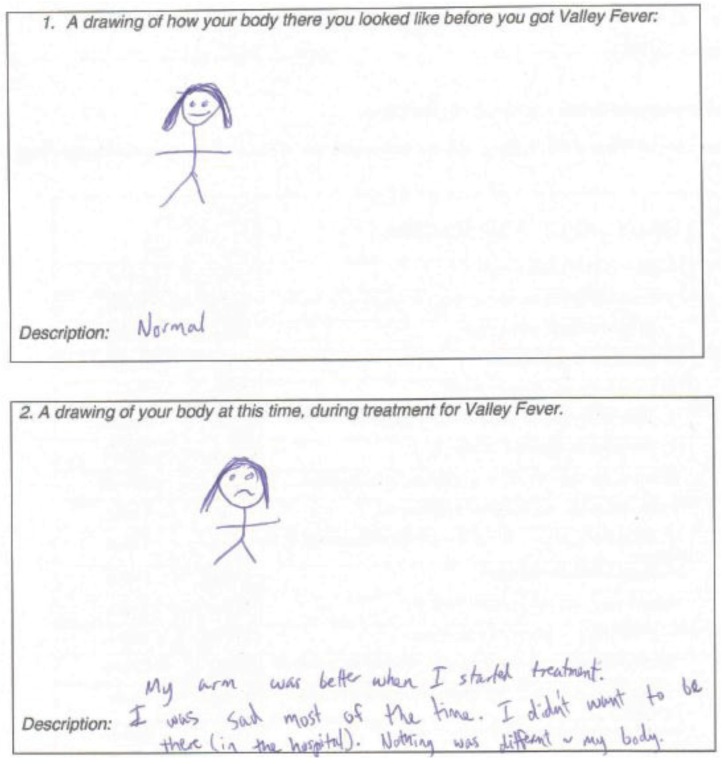
Drawing of a child with coccidioidomycosis with an asymptomatic presentation.

Each child viewed their coccidioidomycosis differently. However, several drawings appeared to reflect children’s focus on physical symptoms (Figure 3). Those who did not focus on symptoms most commonly illustrated and later narrated drawings that were relatively similar before diagnosis and during treatment (Figure 4). This was consistent with child and parent reports of some children who did not understand why they were hospitalized. Some children even went so far as to express resentment at their hospitalizations which kept them from their friends.

### 3.6. Children’s Illness Perceptions

Each patient gave information about their illness beliefs concerning the timeline, control/cure, consequences, and causes of their illness. Notably, every child questioned (15) believed that their illness would improve in time. However, close to half of the children (6) believed that their illness would last a short time and several (5) believed that their valley fever was “likely to last forever rather than go away soon”. Most children (11) believed that coccidioidomycosis is “a serious illness”, though most (10) also reported that it “has become easier to live with”. About half of the children (7) indicated that they believed that their illness “strongly affects the way others think about” them and how they think of themselves as a person (8). Half also reported that their illness cost their families “a lot of money”. Many (10) of them believed that they could control how valley fever affected them and (11) stated that the treatment would help cure them, despite a majority (9) also reporting that “there is little [they can] do to make [their] illness better”.

Children attributed the cause of valley fever to every category on the CIPQ, including stress, family history, diet, bad medical care, their own behavior, family problems or worries, and how they felt. However, the majority believed that valley fever was caused by a germ (8), chance (10), and/or pollution (11), indicating that many children did not have a clear understanding of what caused their illness.

### 3.7. Children’s Coping Strategies

**Figure 5:**
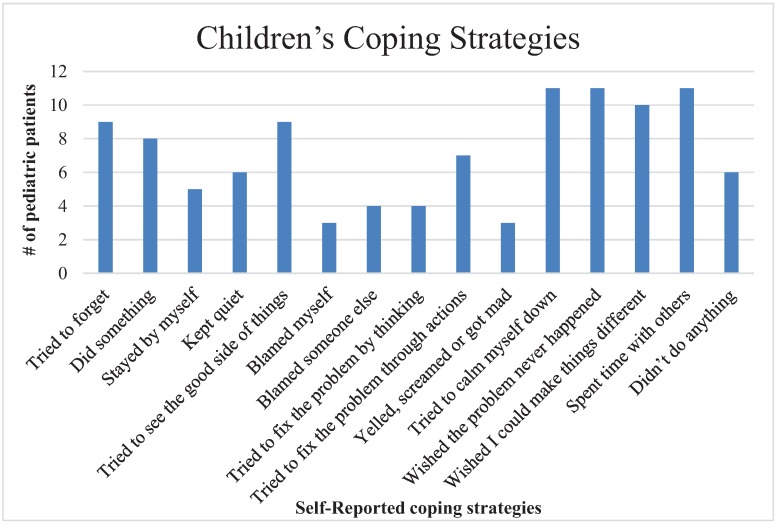
Self-reported coping skills of children with coccidioidomycosis.

Children tended to use varying amounts of the coping strategies of cognitive restructuring, distraction, emotional expression, problem-solving, resignation, self-criticism, social support, social withdrawal, and wishful thinking to cope with valley fever. The frequencies of these strategies are illustrated in Figure 5.

## 4. Discussion

This is the first psychosocial research study conducted on pediatric (or any) coccidioidomycosis patients. Children and their families expressed many concerns that might be addressed by researchers, caregivers, and healthcare providers. However, the data is drawn from a small sample and must be interpreted with caution. Additionally, the child and adult participants were not a homogenous group. Diversity factors such as age and culture may influence some of the factors explored (such as illness perceptions and coping styles). Although the various tools incorporated in the child questionnaires were designed specifically for children and administered by a pediatric health psychologist (the PI), the children’s developmental levels ranged, affecting their responses to the topics. Child participants also varied in terms of the severity of their illnesses. Although English to Spanish back translation methods were used in both the design and data collection phases of the research, meanings are inevitably altered slightly during the translation process. Nonetheless, the passion with which many of the families spoke was indicative of its saliency and the need for further inquiry in this area.

Overall, the psychological functioning, quality of life, and illness perceptions of pediatric coccidioidomycosis patients and their families are difficult to interpret due to the diversity of responses. Many of the perspectives of those with severe coccidioidomycosis symptoms mirror those of pediatric patients of many other long-term illnesses. For example, children with cardiovascular disease [32] and asthma [33] perceive lower quality of life than healthy children, often due to symptom burden and emotional strain. Parents of children with other illnesses frequently cite concern about the illness, treatment and difficulty relieving their children’s symptoms, as is the case with pediatric coccidioidomycosis patients. However, it is rare for children illnesses of equal severity (according to doctors recording titers) to vary so greatly in terms of symptom burden. A comparison study of patient symptoms, fungal titers, and quality of life is needed to test this observation. The finding that many of the child participants did not have a clear understanding of what caused their illness is not surprising, considering that this concept is influenced by children’s ages [34] and parental/family level of understanding, which was also often inaccurate. Complications from the patient and healthcare providers’ perspectives of detection, access to care and treatment further complicate this illness, which receives far less attention than non-orphan diseases.

In caring for the psychosocial needs of children with coccidioidomycosis, coping skills could be taught for dealing with symptoms such as commonly-reported fatigue and fever. Coping involves changing cognitions and/or behavior to control, lessen or endure stressful conditions [35]. Stress management programs for children and their families which include improving control beliefs and self-esteem have been found to improve outcomes for children [36,37]. Rather than allowing families to continue helpless behaviors (such as children “feeling so bad, [they] didn’t want to do anything”), more helpful behaviors could be encouraged, such as breathing and exercise (when medically-appropriate) as distractions from low-level symptoms and social support as a longer-term coping mechanism. Many children in this sample were able to focus on “the good side of things” and distract themselves with social or other activities. Enabling caregivers to help their children either divert their attention or address symptoms could improve the wellbeing of the whole family, which appears altered by most cases of coccidioidomycosis. Educating families (especially about the cause, treatment, and potential psychosocial effects of coccidioidomycosis on their child) in the language they communicate in most effectively might be needed. Most primary caregivers indicated that they had received most of the information they needed, but had many unanswered questions about the treatment and the emotional and social effects of coccidioidomycosis on their child. During and after the interviews and surveys were completed, primary caregivers often asked a multitude of questions. The most frequently asked questions were compiled, answered, and sent back to all participants on the completion of the study with thank you notes and an invitation to participate in “Valley Fever Awareness Day”, an educational public health intervention which took place on the University of California, Merced campus. These questions have been listed in Box 1. Other health interventions supported by medical doctors and public health personnel could help diffuse many of the misconceptions about the illness identified in this research.

It is clear that coccidioidomycosis is a mysterious illness. The wide range of symptoms (ranging from almost none to extremely concerning) make the illness difficult to classify. Those who express fewer symptoms may have more difficulty adhering to treatment and coping with the disease. When symptoms are not noticeable or uncomfortable but require treatment, patients may not adhere to prescribed treatments because they do not believe they are sick or in order to avoid negative side effects. This is not unique to coccidioidomycosis, but is complicated by its sometimes paradoxical presentation (some children may have high titers with very few physical symptoms). Illness beliefs and symptoms may be examined in relation to adherence to medication in greater depth in future studies.

Children who drew themselves looking almost identical before diagnosis and during treatment (perhaps indicating that they did not feel any different) may not be as likely to comply with treatment or recover as quickly as might be hoped for. This is understandable since they may not understand why they are being hospitalized and resent the seemingly senseless isolation from their friends. The portion of primary caregivers who stated that their children’s health was “good” at the worse time during their children’s treatment may also need further education to understand the implications of having coccidioidomycosis. However, almost all of the participating families believed that coccidioidomycosis is “a serious illness”.

Although coccidioidomycosis is rare on a global scale, its effects are wide-reaching. About half of the children interviewed indicated that they believed that their illness strongly affected the way others thought about them and how they thought of themselves. This may have major implications for their psychosocial and cognitive development. Coccidioidomycosis patients’ perceived quality of life should be monitored since they may require support to maintain self-esteem and healthy relationships. Many children with coccidioidomycosis have experienced the need to try to calm themselves down, and tried to feel better by spending time with others, so many clearly have the resources to help themselves at least some of the time.

Though many primary caregivers believed that their children were exposed to coccidioidomycosis in agricultural settings, a large number did not know where the child was exposed. Since many believed that their children were “not at all likely” to have developed coccidioidomycosis from construction sites (which are some of the most likely places to contain the fungus), education may be warranted for dispelling myths such as coccidioidomycosis coming from pollen and plant matter. It may also benefit the community to inform families to ask a doctor to test specifically for coccidioidomycosis, since most participants indicated that doing so is “very important”.

**Box 1. Frequently Asked Questions from Parents**

***Is there a cure for Valley Fever?***

No. Researchers in the U.S. are currently working on vaccines. Studies are being conducted on a new medication that shows potential for a cure in the future.

***Will my child be on medications for the rest of their life?***

The duration of time that the medication is needed for depends on the severity of the disease and your child’s response. Children with Central Nervous System involvement will stay on medications for life. Children with vertebral (spine) disease will stay on medications until they are adolescents (teens).

***Can the medication be changed to improve my child’s health?***

Your doctor will let you know which medication is best for your child depending on the severity of their disease and how your child’s body might react to medication.

***How will Valley Fever affect the development of my child?***

How your child’s body is affected depends on which body system the disease starts in. When the disease is severe and affecting the Central Nervous System, the child’s neurological system may be damaged by valley fever.

***Will Valley Fever affect my child’s ability to have children?***

We don’t know if valley fever affects pregnancy. One of the medications used to treat valley fever (fluconazole) can affect the baby of pregnant women who take it, though. Children taking Fluconazole should not get pregnant while on the medication.

***Will Valley Fever affect children my child may have in the future?***

Valley Fever in newborns is rare. Most babies who acquire Valley Fever do so by inhaling the spores from the environment. The disease is not contagious person to person.

***Would it best for my child to move to another state where the endemic is not severe?***

This depends on the severity of the disease and many other factors. Please ask your doctor.

***Are there any side effects to the Valley Fever medication my child is taking? And if so, what are they?***

This depends on the medication your child is taking. If your child is on Fluconazole, please look out for hepatic dysfunction (liver problems which are not very common). Please ask your doctor.

***After receiving treatment is it possible that my child is still experiencing symptoms of Valley Fever? What can I do about it?***

Symptoms should improve while your child is on medication, but if they do recur, please inform the doctor who tested your child for valley fever. Please tell your child’s new doctor about valley fever if they change doctors, especially if you move outside Central California.

***Are there different types of Valley Fever?***

Yes. Valley Fever can affect different body systems in different patients. There are two different fungi which cause Valley Fever. The one which affects most of the patients in California is called “cocci immitis”. The fungus that causes infection in most places outside California is called “cocci posadasii”.

***What symptoms should I be looking out for and bring to my doctor’s attention?***

If any of your child’s current symptoms get worse or your child develops a fever, cough, headache, rash, or muscle or joint pain, please tell your doctor. In severe cases, valley fever can develop into chronic pneumonia (lung infection) or meningitis (spine or brain infection) or infect bones and joints.

***What are the long term effects of having Valley Fever?***

Since we have not known about valley fever for very long, we do not have information about the long-term effects of childhood valley fever. Your continued participation in research projects like this one will help us help families in the future.

## 5. Conclusions

Science and medical providers may benefit from an improved understanding of psychosocial impact in children diagnosed with coccidioidomycosis who are receiving hospital services. Benefits to the population from information about families’ experiences with coccidioidomycosis include improved care including psychosocial and educational interventions provided to coccidioidomycosis patients. This improved knowledge within the medical and pediatric community in the San Joaquin Valley, in turn, may result in greater public awareness of the illness and better investment of resources.
